# Endoplasmic Reticulum Stress and Lipid Metabolism: Mechanisms and Therapeutic Potential

**DOI:** 10.1155/2012/841362

**Published:** 2011-12-13

**Authors:** Sana Basseri, Richard C. Austin

**Affiliations:** Division of Nephrology, Department of Medicine, McMaster University and St. Joseph's Healthcare Hamilton, 50 Charlton Avenue East, Hamilton, ON, Canada L8N 4A6

## Abstract

The endoplasmic reticulum (ER) plays a crucial role in protein folding, assembly, and secretion. Disruption of ER homeostasis may lead to accumulation of misfolded or unfolded proteins in the ER lumen, a condition referred to as ER stress. In response to ER stress, a signal transduction pathway known as the unfolded protein response (UPR) is activated. UPR activation allows the cell to cope with an increased protein-folding demand on the ER. Recent studies have shown that ER stress/UPR activation plays a critical role in lipid metabolism and homeostasis. ER-stress-dependent dysregulation of lipid metabolism may lead to dyslipidemia, insulin resistance, cardiovascular disease, type 2 diabetes, and obesity. In this paper, we examine recent findings illustrating the important role ER stress/UPR signalling pathways play in regulation of lipid metabolism, and how they may lead to dysregulation of lipid homeostasis.

## 1. Introduction

The liver plays a central role in whole body lipid homeostasis. Metabolic signals such as carbohydrates and dietary fatty acids regulate hepatic gene expression leading to glycolytic and lipogenic signalling pathways. In addition, the pancreatic hormones, insulin and glucagon, play a pivotal role in the transcriptional and posttranslational regulation of lipogenesis and lipid oxidation [1]. Lipogenesis, the process of *de novo *lipid biosynthesis, occurs when an excess of carbohydrates is consumed, or when circulating insulin levels are high. Carbohydrates undergo glycolysis to generate acetyl-CoA molecules which are the building blocks for fatty acid (FA) synthesis. Following esterification, one glycerol molecule and three FA chains produce triacylglycerol (TG) molecules which are transported in apoB containing very low-density lipoprotein (VLDL) particles [2] to the adipose tissue for long-term storage. Under fasting conditions when insulin levels are low and glucagon levels are high, FA oxidation or lipolysis occurs which allows for mobilization of FA and uptake by the liver [3]. However, disruption in these homeostatic mechanisms may lead to the development of dyslipidemia, insulin resistance, fatty liver, and excess adipose mass, ultimately causing cardiovascular disease and diabetes.

In recent years, increasing evidence suggests that ER stress and UPR activation can regulate cellular processes beyond ER protein folding and can play crucial roles in lipid metabolism [4–10]. ER stress, which occurs due to disruption in ER protein-folding capacity, leads to activation of an evolutionarily conserved UPR signalling system in order to restore ER homeostasis [11]. Accumulating evidence suggests that activation of the UPR pathways can modulate lipid metabolism by controlling the transcriptional regulation of lipogenesis. Excess adipose mass and obesity are a direct consequence of increased *de novo *lipogenesis and TG storage in the adipose tissue. The presence of ER stress has been observed in various tissues from obese mice [12, 13] and humans [14–17]. UPR activation has also been linked to fatty liver disease where lipid droplets accumulate in hepatocytes. The role of ER stress and UPR pathways in the development of fatty liver disease has been under intense investigation (reviewed in [18]). Here, we aim to examine the evidence regarding the role of UPR pathways in modulating the transcriptional regulation of lipid metabolism. Furthermore, potential therapeutic approaches targeting the ER stress response in obesity and dyslipidemia will be discussed.

## 2. Transcriptional Regulation of Lipid Metabolism

A number of key transcription factors have been identified which regulate hepatic lipogenesis and fatty acid oxidation. These include sterol-regulatory-element-binding protein-1c (SREBP-1c), liver X receptor (LXR), peroxisome-proliferator-activated receptors (PPARs), and carbohydrate-responsive-element-binding protein (ChREBP). Enzymes such as glucokinase (GK), liver pyruvate kinase (LPK), acetyl CoA carboxylase (ACC), fatty acid synthase (FAS), and stearoyl CoA desaturase-1 (SCD-1) are critical for the biochemical conversion of glucose into fatty acids and TG [19, 20].

SREBP-1c, a member of the SREBP family of transcription factors, is thought to be the main driving force for hepatic lipogenesis and development of fatty liver disease known as hepatic steatosis [21, 22]. There are three isoforms, SREBP-1a, -1c and -2. SREBPs are synthesized as inactive precursors bound to the ER membrane [23]. While SREBP-1a/-2 upregulate cholesterol synthesis genes, SREBP-1c is responsible for the regulation of genes involved in FA and TG synthesis pathways [24]. Under sterol-replete conditions, SREBPs are held in the ER through their interaction with SCAP, an anchoring molecule, and Insig, an ER transmembrane protein. The SREBP-SCAP complex is released from Insig upon sterol-deplete conditions. SCAP assists in the transport of SREBP from the ER to the golgi for cleavage by site 1 and 2 proteases [25]. Following proteolytic cleavage, the active mature form of SREBP translocates into the nucleus where it induces genes required for lipid biosynthesis and uptake [26].

SREBP-2 is the main transcription factor responsible for regulating the cholesterol biosynthetic pathways [27]. Cholesterol is the precursor for steroid biosynthesis and plays an important role in membrane biology. Excess unesterified intracellular cholesterol can lead to membrane disruptions and cellular toxicity and hence must be tightly regulated [28]. Therefore, under sterol-deplete conditions, SREBP-2 is cleaved and translocates to the nucleus allowing for expression of its target genes, including HMG-CoA reductase, the rate-limiting enzyme in cholesterol biosynthesis [29, 30].

SREBP-1c is the predominant isoform and the main regulator of lipid synthesis in the liver [31]. Overexpression of the active form of SREBP-1 in the liver leads to hepatic steatosis due to increased lipid synthesis, uptake, and TG accumulation [32], while loss of SREBP-1 has been linked to marked reduction in both lipogenesis and hepatic steatosis [22, 33]. Interestingly, the proteolytic cleavage of SREBP-1c is not affected by sterol depletion [34]. Proteolytic cleavage and activation of SREBP-1c is stimulated by insulin [35]. Insulin-mediated SREBP-1c activation occurs through insulin receptor substrate-1 (IRS1) and activation of its downstream targets protein kinase B (PKB/Akt) and mammalian target of rapamycin complex 1 (mTORC1) [34]. Although the exact mechanism by which insulin stimulates SREBP-1c cleavage is not entirely understood, it has been shown that insulin leads to phosphorylation of the ER-bound inactive SREBP-1c, increasing its posttranslational processing [36]. Furthermore, insulin represses Insig2 mRNA which is thought to enhance SREBP-1c activation [37, 38].

SREBP-1c activity may also be induced through the nuclear hormone receptor peroxisome-proliferator-activated receptor-*γ* (PPAR*γ*) [39] as well as liver X receptor (LXR) activity [20], both of which play a critical role in lipogenesis. Ligand-activated nuclear PPAR*γ* heterodimerizes with retinoid X receptors (RXRs) resulting in expression of its target genes such as CD36, a fatty acid transport protein involved in the transport and metabolism of intracellular FA [40]. Ultimately, PPAR*γ* activity allows for transcription of genes involved in promoting lipogenesis [41]. In addition, positive feedback loops have been identified where SREBP-1c activity increases the formation of PPAR*γ* ligands which lead to its activation [42]. PPAR*γ* also leads to LXR*α* gene expression which is a potent activator of SREBP-1c target genes [40]. PPAR*α* activity, on the other hand, regulates peroxisomal, microsomal, and mitochondrial FA oxidation pathways by transcriptionally regulating enzymes involved in these pathways [40]. Interestingly, LXR competes with PPAR*α* for RXR*α* heterodimerization, thereby repressing RXR*α*-PPAR*α* signalling. This in turn suppresses LXR-SREBP-1c activity [40]. This crosstalk would ensure that lipogenic and lipolytic pathways are not simultaneously activated. Finally, lipogenic and glycolytic gene expression may also be regulated by ChREBP, a transcription factor responsive to high glucose levels and important in regulating the expression of LPK, an enzyme required for hepatic glycolysis [43].

 In addition to regulation of lipogenic and lipolytic pathways, fatty acid uptake and lipoprotein secretion are also important for lipid homeostasis. Expression of PPAR*α*, for example, leads to mobilization and transport of catabolized fatty acids by inducing expression of enzymes such as fatty-acid-binding protein (FABP) and fatty-acid translocase (FAT) [40]. Fatty acids undergo esterification to form TG which can be exported out of the liver as VLDL particles. ApoB is the key component of VLDL particles and microsomal triacylglycerol transfer protein (MTP) allows for the addition of TG to apoB, forming the VLDL particle. However, the overall rate of VLDL assembly depends on the rate of apoB synthesis in the ER [40].

## 3. The ER and UPR Activation

The ER is a membranous organelle with several critical cellular functions. First, it is the site where nascent polypeptides fold into their proper conformation and any necessary posttranslational modifications such as glycosylation and disulphide bond formation take place. This task is accomplished by ER resident chaperones and foldases and protein disulphide isomerases (PDI) [11]. Second, phospholipid synthesis takes place in the ER which allows for expansion of lipid bilayers in the cell [1]. Third, the ER is a major storage site for calcium ions which are required for cellular signalling processes [44]. Fourth, enzymes such as cytochrome p450 in the ER allow for efficient metabolism of drugs [45].

A number of physiological, pharmacological, and pathological conditions are known to disrupt ER homeostasis and affect its protein-folding capacity. The inability of the cell to efficiently fold and secrete proteins is defined as ER stress. Cells have evolved mechanisms to adapt to adverse conditions in order to maintain homeostasis and survive. One such coping mechanism is UPR activation in response to ER stress conditions [46, 47]. Activation of the UPR ultimately results in (i) enhancement of ER protein-folding capacity through expansion of the ER and increased expression of chaperones and foldases, (ii) inhibition of protein translation, and (iii) ER-associated protein degradation (ERAD) of misfolded proteins [48]. If ER stress conditions are not resolved, ER-stress-induced cell death may ensue. Generally, ER-stress-associated cell death occurs through caspase activation [49, 50]; however, caspase-independent necrosis and autophagy have also been observed [51].

**Figure 1:**
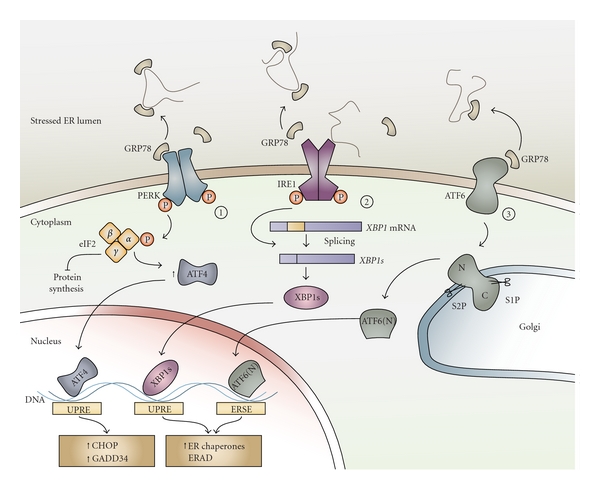
ER stress and activation of the UPR signalling pathways. Accumulation of misfolded or unfolded protein aggregates in the ER lumen, a condition known as ER stress, leads to activation of three ER transmembrane proteins, PERK, IRE1, and ATF6. GRP78, a ubiquitous ER chaperone that is normally bound to these ER stress sensors and keeps them inactive, dissociates from them in order to assist with the folding of proteins in the ER lumen. However, this dissociation leads to activation of the 3 UPR pathways. (1) PERK homodimerization and autophosphorylation results in the subsequent phosphorylation of the *α* subunit of eIF2 which by inhibiting global protein synthesis reduces the ER protein load. ATF4 expression, however, increases upon eIF2*α* phosphorylation which translocates to the nucleus allowing for transcription of UPR target genes by binding to the UPR response element (UPRE). These genes include CHOP, a proapoptotic transcription factor that results in cell death if ER stress conditions persist, and GADD34, which acts as a negative regulator of the PERK pathway by dephosphorylating eIF2*α*. (2) IRE1 is activated in a similar manner to PERK by homodimerization and autophosphorylation. Additionally, interaction of misfolded or unfolded proteins with the luminal domain of IRE1 can also further promote its activation. *XBP1* mRNA is an IRE1 substrate that undergoes splicing to produce *XBP1s*, encoding a transcription factor that can lead to upregulation of ER chaperones and other UPR target genes. (3) ATF6 activation leads to its translocation to the Golgi where it is sequentially cleaved by site 1 and site 2 proteases. This leads to the release of the N-terminal ATF6 fragment which translocates to the nucleus, binds to the ER stress response element (ERSE) thereby activating UPR target genes.

The UPR in mammalian cells is composed of three signalling branches which are initiated by three ER transmembrane sensors, inositol-requiring protein 1 (IRE1), double-stranded RNA-dependent protein kinase-like ER kinase (PERK), and activating transcription factor 6 (ATF6). Activation of these sensors is dependent on the dissociation of the ER-resident chaperone glucose-regulated protein of 78 kDa (GRP78), also known as BiP, from their luminal domains [52]. This occurs during ER stress conditions when GRP78 is required for the folding of proteins in the ER and thus is recruited away from IRE1, PERK, and ATF6, thereby activating the UPR. Activation of the UPR pathways is often used as an indicator of ER stress due to the technical difficulties in directly measuring compromised ER integrity or protein aggregates in the ER [1].  Figure 1 depicts an overview of mammalian UPR signalling pathways.

Homodimerization and autophosphorylation of PERK following dissociation of GRP78 leads to its kinase activity. PERK phosphorylates the *α* subunit of eukaryotic initiation factor 2 (eIF2) resulting in translational attenuation [53]. Translation of certain mRNAs with short open reading frames in the 5′-UTR is enhanced by phosphorylation of eIF2*α*. ATF4 is an example of such mRNA and its expression results in activation of C/EBP homologous protein (CHOP) which is a proapoptotic transcription factor [54]. GADD34 (growth-arrest and DNA-damage-inducible protein 34) is induced by CHOP, which acts to dephosphorylate eIF2*α* as a negative feedback loop and relieve the cell of the translational repression during prolonged ER stress [55].

Similar to PERK, IRE1 is a type 1 transmembrane serine/threonine receptor protein kinase/endonuclease which upon dissociation of GRP78 homodimerizes leading to autophosphorylation and activation of its kinase and endoribonuclease functions [48]. Unfolded proteins may also directly bind to IRE1 promoting its homodimerization and autophosphorylation [56–58]. Activation of IRE1 results in splicing of *XBP1 *mRNA, a process by which a 26-nucleotide sequence of *XBP1* mRNA is excised leading to a shift in its reading frame. Unlike the unspliced XBP1 protein, which is rapidly degraded, spliced *XBP1* (*XBP1s*) encodes a bZIP transcription factor with a potent transactivation domain [59]. *XBP1s* translocates to the nucleus where it leads to expression of a number of UPR target genes including genes involved in protein folding and secretion, protein degradation and ER translocation [1, 60]. Consistent with its transcriptional target genes, *XBP1* is required for the secretory function of certain highly secretory cell types such as antibody-producing plasma cells [61].

ATF6, the third arm of the UPR, is comprised of two transmembrane bZIP transcription factors, ATF6*α* and ATF6*β*, which under normal conditions are held in the ER in a complex with GRP78 [62]. ER stress and dissociation of GRP78 from ATF6 leads to its translocation to the Golgi where it is cleaved by site 1 and site 2 proteases, a process similar to that of the SREBPs. The sequential proteolysis by S1P and S2P leads to the release of the N-terminal cytosolic domain of ATF6 which then upon entry into the nucleus activates UPR target genes [63]. Among these target genes are XBP1, CHOP, and ER chaperones such as GRP78 which allow the ER to cope with the increased protein-folding demand [62, 64]. Interestingly, ATF6 and XBP1 possess very similar DNA-binding specificity [60] and can heterodimerize suggesting that they may have common target genes [65].

## 4. ER Stress and Lipid Metabolism

**Figure 2:**
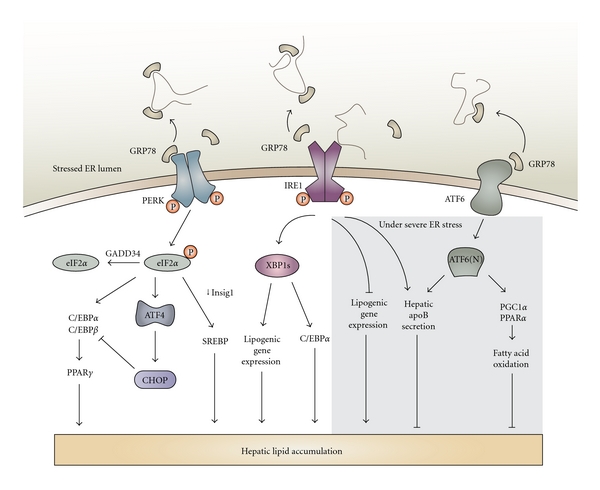
Crosstalk between UPR signalling pathways and lipogenesis. Phosphorylation of eIF2*α* and activation of the PERK pathway under high-fat diet-induced ER stress conditions allow for enhanced lipogenesis by inducing C/EBP*α* and decreasing Insig1 protein translation which increases activation of SREBP. However, under severe or prolonged ER stress conditions, CHOP expression may lead to dysregulation of the C/EBPs. Similarly, high-carbohydrate-diet-induced ER stress conditions depend on XBP1 for expression of lipogenic genes and increase of C/EBP*α* activity, both of which promote lipogenesis. However, severe ER stress conditions, imposed by tunicamycin, lead to XBP1-mediated inhibition of lipogenic gene expression. Furthermore, both XBP1 and ATF6 are important for apolipoprotein B secretion from hepatocytes and activation of fatty acid oxidation pathways (PPAR*α*, PGC1*α*) under such conditions. These pathways culminate in attenuation of lipogenesis and prevention of fatty liver disease under severe ER stress.

It is has been known for about a decade that ER stress can lead to altered lipid metabolism and hepatic steatosis. A study by our group demonstrated that homocysteine-induced ER stress can lead to hepatic steatosis and altered cholesterol and TG biosynthetic pathways, both in cultured cells and in livers of hyperhomocysteinemic mice [66]. Overexpression of GRP78, which attenuates ER stress and UPR activation, has been shown to decrease hepatic steatosis by reducing SREBP-1c activity [5]. More recently, specific arms of the UPR and their downstream signalling molecules have been examined in cell culture and animal models to decipher their function and role in lipid metabolism. It is now well established that various components of the UPR signalling network play a role in the regulation of lipid metabolism [4–10].  Figure 2 summarizes the interactions between various components of UPR signalling and lipid metabolism.

### 4.1. PERK Pathway

Activation of PERK is transient and has often been difficult to detect [10], but recently the Phos-tag gel approach has proven to be a successful tool for detection of PERK phosphorylation [67]. Furthermore, the phosphorylation status of eIF2*α*, a downstream target of PERK, is often assessed to monitor PERK activity. Changes in nutritional status such as fasting and feeding result in altered phosphorylation status of eIF2*α*. Fasting followed by 4 hours of feeding leads to an increase in phospho-eIF2*α* levels in the liver, which were even greater in high-fat-diet-fed mice [10]. To study the effects of compromised PERK-eIF2a-dependent UPR signalling, transgenic mice with enforced expression of GADD34 were generated [10]. GADD34, by associating with protein phosphatase 1, acts to specifically dephosphorylate eIF2*α*. Therefore, these mice were defective in activating the gene expression program downstream of eIF2*α* phosphorylation upon feeding and under severe ER stress conditions [10]. Close examination of the metabolic changes in the transgenic mice indicated that defective eIF2*α*-mediated signalling results in fasting hypoglycemia, reduced liver glycogen stores, and enhanced insulin sensitivity. Additionally, under dietary stress of a high-fat diet, the transgenic mice exhibited reduced hepatosteatosis and greater insulin sensitivity as compared to wild-type mice [10]. Expression of PPAR*γ* and its lipogenic target genes was reduced in the transgenic mice with the eIF2*α* phosphorylation defect only when fed a high-fat diet. Repressed expression of C/EBP*α* and C/EBP*β* proteins was also observed in livers of transgenic mice [10].

Rutkowski et al. generated mice harbouring a S51A mutation in eIF2*α* rendering them unable to phosphorylate eIF2*α* and therefore allowing constitutive expression of the unphosphorylated form of eIF2*α* [7]. This transgenic mouse model was also utilized to examine the PERK/eIF2*α* arm of the UPR and its role in ER-stress-mediated hepatic lipogenesis [7]. Similar to the findings by Oyadomari et al., mice with constitutive eIF2*α* expression also exhibited suppressed hepatic C/EBP*α* protein expression. In contrast, however, after a tunicamycin challenge, these mice developed fatty liver [7]. These differences point to the source and severity of ER stress (chronic and adaptive dietary stress versus direct and acute ER stress challenge) as important factors in the regulation of lipid metabolism.

Another recent study examined the role of PERK in the regulation of lipogenesis in adipocytes and mammary epithelial cells [4]. Absence of PERK in mouse embryonic fibroblasts differentiating into adipocytes and in mammary epithelium attenuated lipogenesis and expression of genes such as *SREBP1*, *SCD1*, *FAS*, and *ACL* [4]. As a result, the mammary glands from PERK-deficient mothers had lower TG and FA content which lead to growth retardation in the pups. This study also demonstrated that SREBP1 activation is dependent on decreased Insig1 translation which occurs due to PERK and eIF2*α*-dependent translational attenuation [4].

Due to its upstream open reading frames, the *ATF4* mRNA is among the transcripts that escape the global translational attenuation that occurs upon phosphorylation of eIF2*α*. ATF4-knockout mice exhibit smaller white adipose tissues relative to total body weight [68], which prompted a closer examination of these mice. Yoshizawa et al. revealed that ATF4 alters glucose metabolism by decreasing insulin sensitivity in the liver, adipose and muscle tissue [68]. Wang et al. reported decreased expression of lipogenic genes and increased beta-oxidation in the white adipose tissue of ATF4-knockout mice [69]. Interestingly, these observations were not reproducible in primary cell cultures which led to the identification of osteoblastic ATF4 expression as the regulator of whole-body energy homeostasis [68]. Taken together, these results suggest that the PERK-eIF2*α* pathway plays an important role in promoting lipogenesis both in the liver and other tissues.

### 4.2. IRE Pathway

A recent study by Zhang et al. demonstrated that IRE1*α* has an important role in preventing ER stress-induced hepatic steatosis [9]. Given that *Ire1*α**-null mice die during embryogenesis, hepatocyte-specific *Ire1*α**-null (*Ire1*α** ^Hepfe/-^) mice were generated to understand the function of IRE1*α* in hepatocytes. These mice appeared phenotypically normal in the absence of a stress challenge. However, treatment of *Ire1*α** ^Hepfe/-^ mice with tunicamycin, an ER-stress-inducing agent that inhibits protein N-glycosylation [46], led to identification of a defective adaptation to ER stress and altered lipid metabolism in the absence of IRE1*α* [9]. Expression of ER stress-induced proapoptotic transcription factors ATF4, CHOP, and ATF3 were increased in tunicamycin treated *Ire1*α** ^Hepfe/-^ mouse livers as compared to control mice. The number of TUNEL-positive apoptotic cells and cleaved caspase-3 expression was also higher in *Ire1*α** ^Hepfe/-^  livers [9]. Furthermore, evaluation of hepatic fat content and plasma lipids revealed that *Ire1*α** ^Hepfe/-^ livers have enhanced hepatic steatosis and reduced plasma lipids due to suppressed apoB-containing lipoprotein secretion. Increased expression of key lipogenic transcription factors such as PPAR*γ*, C/EBP*β*, ChREBP, and LXR*α* and greater expression of mRNA encoding lipogenic enzymes such as SCD1, DGAT2, DGAT1, and ACC1 were observed in *Ire1*α** ^Hepfe/-^ livers, in particular after tunicamycin treatment [9]. Taken together, these findings suggest that IRE1*α* is required to suppress hepatic lipid accumulation, particularly under severe ER stress conditions.

A report by Iqbal et al. demonstrated that IRE1*β* may also have an important role in lipid metabolism primarily in intestinal cells [70]. *Ire1*β**−/− mice fed a high-fat and high-cholesterol diet developed hyperlipidemia due to enhanced microsomal triglyceride transfer protein (MTP) expression in enterocytes which led to increased chylomicron secretion [70].

Interestingly, XBP-1 a transcription factor downstream of IRE1 activation has a role in hepatic lipid regulation independent of being an ER stress-response mediator [6]. *Xbp1-*null mice die during embryogenesis; however, deletion of XBP1 in the liver led to hypodyslipidemia and reduced expression of genes encoding lipogenic enzymes such as DGAT2, SCD1, and ACC2 [6]. Livers from mice with an XBP1 deletion had diminished hepatic TG secretion and lipid synthesis but the rate of apoB protein turnover was not affected [6]. These findings indicated that XBP1 is required for *de novo* lipid synthesis in the liver. While liver XBP1 deficiency did not itself cause ER stress or any obvious liver or body abnormalities, there was evidence for increased activation of its upstream kinase IRE1, likely due to a regulatory feedback mechanism [6]. This may in part explain why absence of hepatic IRE1*α* led to increased lipid accumulation, while deficiency in its downstream target XBP1 did not affect steatosis. Increased activation of IRE1 which is required to suppress lipogenesis may be influencing the phenotypic outcome in the mice deficient in hepatic XBP1. XBP1 likely does not regulate ER stress-mediated steatosis as tunicamycin-induced fatty liver occurred both in the presence and absence of spliced XBP1 [7]. There is evidence suggesting that in adipocytes, XBP1 binds to the promoter region of C/EBP*α* which promotes adipogenesis and lipid deposition [71]. XBP1 also plays an important role in phosphatidylcholine synthesis, the main ER membrane phospholipid which allows for ER biogenesis and expansion under ER stress conditions [72].

### 4.3. ATF6 Pathway

ATF6 and SREBPs are ER membrane-bound transcription factors and their activation is dependent on cleavage by the same proteases in the Golgi, followed by nuclear translocation of the N-terminal fragment to the nucleus [63, 73]. ER stress has been linked to the activation and cleavage of both ATF6 and SREBP2 [64, 74, 75]. A close examination of the relationship between ATF6 activity and SREBP2-mediated lipogenesis revealed that nuclear ATF6 interacts with the nuclear form of SREBP2 and thereby antagonizes SREBP2-regulated transcription of lipogenic genes and lipid accumulation in cultured liver and kidney cells [73]. The authors suggest that this negative regulation of SREBP2 activity by ATF6 accumulation in the nucleus would allow the cell to cope with ER stress conditions and save on cellular energy resources.

Several recent studies have examined the role of ATF6 *in vivo *by studying the role of ER stress on fatty liver disease and lipid droplet formation in ATF6*α*-knockout mice [7, 76, 77]. Interestingly, similar to *Ire1*α** ^Hepfe/-^ mice, ATF6*α*-knockout mice exhibited no apparent phenotype under physiological conditions; however, when given an ER stress insult by injection of tunicamycin, the livers in the knockout mice were unable to recover [76, 77]. Livers from tunicamycin-injected ATF6*α*-knockout mice showed signs of dysfunction as measured by serum ALT, protein content, and albumin levels [76]. Furthermore, the livers in the knockout mice had greatly reduced expression of ER chaperones following tunicamycin injection and increased numbers of TUNEL-positive apoptotic cells, suggesting that ATF6 protects hepatocytes from ER stress-induced damage and apoptosis [76]. The differences observed in tunicamycin-injected ATF6*α*-knockout mice as compared to wild-type mice are likely not due to increased cytotoxicity of tunicamycin in the ATF6*α*-knockout mice as no significant differences were noted in the upregulation of cytochrome P450 isoforms and cleavage of nuclear PARP between the groups of mice [7].

The phenotypic outcome of the ER stress insult in ATF6*α*-knockout mice was hepatic steatosis caused by induction of lipid droplet formation due to reduced *β*-oxidation of FA and attenuated VLDL formation [76]. Specifically, there was sustained expression of CHOP in the livers of ATF6*α*-knockout mice compared to wild-type mice as well as a decrease in PPAR*α* expression and apoB-100 protein levels, favouring the accumulation of lipids in the liver [7, 76]. *De novo* lipogenesis was ruled out as a mechanism for the increased lipid droplet accumulation in livers from these mice as expression of lipogenic genes (*SCD1*, *FASN*, and *DGAT2*) was suppressed in tunicamycin-injected ATF6*α*-knockout mouse livers [7]. In addition, while steatosis was the most evident phenotype, upon closer examination it was discovered that after 48 hours of tunicamycin treatment, ATF6*α*-knockout mice became profoundly resistant to exogenous insulin [7]. This finding is intuitive given that ER stress can lead to insulin resistance [12]. Taken together, the findings from these studies suggest that loss of ATF6 predisposes the liver to stress-induced insulin resistance and lipid accumulation.

The studies to date suggest that lipogenic genes and lipid metabolism are differentially regulated under physiological conditions such as high-carbohydrate or high-fat diet feeding in comparison to acute or unresolved ER stress conditions that arise when mice are injected with tunicamycin. For example, while XBP1 increases hepatic *de novo* lipogenesis, its upstream kinase IRE1*α*, or ATF6 which shares DNA-binding sites with XBP1, was protective against hepatic lipid accumulation. Indeed, a recent study by Rutkowski et al. demonstrated that it is the acute or unresolved form of ER stress that leads to hepatic steatosis [7]. Injection of tunicamycin in mice deficient in one of the UPR signalling components led to chronic upregulation of CHOP, defective eIF2*α* phosphorylation, and decreased C/EBP*α* gene expression [7]. CHOP was reported to be at least partially responsible for the suppression of gene expression seen in tunicamycin-injected mice with compromised UPR signalling [7]. While wild-type mice exhibited rapid but transient CHOP induction, ATF6-knockout and *Ire1*α** ^Hepfe/-^ mice presented with persistent upregulation of CHOP and nuclear localization [7]. CHOP can heterodimerize with the C/EBP family of transcription factors in the nucleus repressing their target gene expression [78]. Negative regulation of C/EBP*α* by prolonged nuclear CHOP expression due to unresolved ER stress appears to play a key role in the profound metabolic disruption under severe ER stress conditions which results in fatty liver disease. Indeed, the promoter region of both *Srebp1* and *Ppar*α** possesses potential binding sites for C/EBP*α* [7]. The differential effects of acute/unresolved ER stress conditions in comparison to diet-induced adaptive ER stress conditions also explain why phosphorylation of eIF2*α* can lead to hepatic steatosis in one study model while defective eIF2*α* phosphorylation can accelerate lipid accumulation and steatosis in another study.

## 5. The Impact of Lipids on ER Stress

The relationship between ER stress and lipid metabolism is bidirectional. While activation of ER stress pathways can result in lipogenesis and altered lipid homeostasis, lipids and aberrant lipid metabolism can also cause ER stress [79–82]. Saturated fatty acids such as palmitate and stearate are known inducers of ER stress in various cell types and can modulate survival and apoptotic signals in the cell [81, 82]. A recent study carried out comparative proteomic and lipidomic analysis of fractionated ER from lean and obese liver tissues [79]. The results suggested enrichment of metabolic enzymes involved in lipid metabolism and a downregulation of ER-associated protein synthesis genes in the obese ER proteome. These findings implied that the ER in obese liver cells shifts from being the major site of protein synthesis to carrying out lipid synthesis and lipid metabolism functions [79]. Furthermore, the analysis revealed that there is a greater proportion of *de novo* synthesized saturated fatty acids incorporated into hepatic ER lipids than dietary polyunsaturated fatty acids. Another interesting finding was the increased proportion of phosphatidylcholine (PC) in comparison to phosphatidylethanolamine (PE), both abundant ER membrane phospholipids, in the liver ER from obese mice [79]. The increased PC/PE ratio led to perturbation in the calcium transport activity of the SERCA pump resulting in impaired ER calcium retention. Since ER calcium is important for ER homeostasis and chaperone function, such changes in calcium concentrations would lead to protein misfolding and ER stress. This appears to be a plausible mechanism for hepatic ER stress in obesity [79]. Hepatic ER stress can promote *de novo* lipogenesis and insulin resistance as described above which then in turn may lead to further exacerbation of the ER stress situation, creating a vicious cycle.

## 6. Therapeutic Potential Targeting ER Stress in Dyslipidemia and Obesity

ER stress and UPR activation have been implicated in the pathogenesis of a number of diseases such as diabetes, obesity, cancer, renal, cardiovascular, and neurodegenerative diseases as well as fatty liver disease [48, 83–86]. As such, potential ways of attenuating ER stress and UPR activation would provide opportunities in pharmacological intervention in a wide array of diseases. A recent study revealed for the first time in humans that obese insulin-resistant subjects express markers of ER stress in their white adipose tissue [17]. Similarly, an association between ER stress and obesity was also found in obese nondiabetic subjects [15]. Gastric bypass surgery-mediated weight loss in obese patients was effective at reducing ER stress in adipose and liver tissues and improved insulin sensitivity [16]. Furthermore, when ER stress was reduced by hepatic overexpression of GRP78 in *ob/ob* mice, hepatic TG and cholesterol content was reduced and insulin sensitivity improved [5]. These findings together with data in rodents indicating the presence of ER stress in tissues of obese animals [12, 13] suggest a strong association between ER stress and obesity. Therefore, the ER serves as an important new treatment target against obesity and its metabolic complications.

The use of small molecules called chemical chaperones has been examined in a number of disease models as potential tools for lowering ER stress and preventing the activation of UPR pathways. These chaperones similar to molecular chaperones nonselectively stabilize mutant proteins and assist in their folding and translocation across membranes [87]. Most chemical chaperones are osmolytes and equilibrate cellular osmotic pressure. These can be categorized into 3 classes: carbohydrates (such as glycerol and sorbitol), amino acids (such as glycine and taurine), and methylamines (such as betaine) [87, 88]. The drawback to the use of most chemical chaperones is their nonspecificity and high-dose requirement for effective protein folding properties. However, two such chemical chaperones, 4-phenylbutyric acid (4-PBA) and tauroursodeoxycholic acid (TUDCA) have been approved by the US Food and Drug Administration (FDA) and are used in humans. Currently, 4-PBA is approved for use in children with urea-cycle disorders as an ammonia scavenger, while TUDCA is being tested for its liver-protecting properties in cholestatic liver disease in humans [87].

The low-molecular-weight fatty acid 4-PBA has been tested in a number of disease models for its ability to facilitate protein folding and trafficking, ultimately relieving ER stress [13, 89–98]. The chaperoning property of 4-PBA was first identified when investigating its effect on the translocation and trafficking of a mutant cystic fibrosis transmembrane conductance regulator protein (CFTR). Addition of 4-PBA to the cells allowed for stabilization of the mutant CFTR protein and facilitated their translocation to the cell membrane [99]. In addition to its chaperone properties, 4-PBA also possesses HDAC inhibitor activity and is under investigation as an anticancer drug [100–102].

Another effective reagent that has been shown to have chaperone properties is TUDCA, which can be classified as a hydrophilic endogenous bile acid [87]. TUDCA has antiapoptotic properties by reducing calcium efflux and blocking ER-stress-mediated caspase-12 activation [103]. Furthermore, TUDCA also activates cell survival pathways such as PI3K signalling, thereby inhibiting cell death [104]. Apart from these signalling properties, TUDCA can interact with the mineralocorticoid receptor and promotes its dissociation from cytosolic chaperones thereby preventing its translocation to the nucleus for transcriptional activity. In the case of primary neurons, addition of TUDCA was effective at preventing amyloid beta-peptide-induced apoptosis through its chaperoning properties [105].

In recent years, several studies have identified beneficial effects of 4-PBA and TUDCA supplementation on insulin resistance, obesity, and diabetes. Oral administration of 4-PBA and TUDCA to obese and insulin-resistant *ob/ob* mice normalized hyperglycemia, restored insulin sensitivity in the liver, muscle, and white adipose, and diminished fatty liver disease [92]. Our group examined the effect of 4-PBA supplementation on diet-induced obesity. For this purpose, C57BL/6 mice were placed on a high-fat diet with or without 4-PBA supplementation in the drinking water. Mice treated with 4-PBA gained significantly less weight, exhibited lower plasma glucose, TG, and leptin levels, and had smaller adipocytes as compared to mice on a high-fat diet alone [89]. Chemical chaperones also have chaperone activity within the central nervous system [13, 106]. Leptin, an adipocyte-derived hormone which acts on hypothalamic neurons to suppress appetite, is important in regulating energy expenditure and body weight [107]. ER stress may be one of the factors resulting in leptin resistance in the brain, as injection of tunicamycin, an ER-stressor-induced hypothalamic ER stress, increased food consumption and weight gain despite elevated blood leptin concentrations [13]. Both 4-PBA and TUDCA were shown to be effective at lowering hypothalamic ER stress and increasing the sensitivity of neurons to leptin, thereby reducing body weight in genetic and diet-induced obesity models [107]. In the context of atherosclerosis, 4-PBA was effective at protecting macrophages against palmitate-induced ER stress and apoptosis in culture [108]. A reduction in ER stress and apoptosis was also observed in the macrophages within the atherosclerotic lesions of mice treated with 4-PBA, which were smaller in size [108]. These findings indicate that 4-PBA treatment can protect cells from the deleterious effects of lipid accumulation on disease progression.

ER stress has been linked to fatty liver disease and liver injury [109, 110]. Lipid-induced ER stress inhibits apoB100 secretion in liver cells promoting the development of steatosis [111]. Treatment of hepatoma cells with 4-PBA leads to the inhibition of lipid-induced ER stress and enhanced apoB100 secretion [111]. Consistent with the studies on macrophages and progression of atherosclerosis, alleviating lipid-induced ER stress in hepatocytes also protects the cells from ER-associated apoptosis [112]. Since hepatocellular injury and damage can lead to progression of fatty liver disease into steatohepatitis [113, 114], blocking ER stress serves as an important treatment strategy [95]. A recent study examined the effects of oral administration of TUDCA on hepatic steatosis and hepatic gene expression in *ob/ob* mice [115]. Yang et al. found a significant decrease in liver fat content and reduced expression of genes involved in *de novo *lipogenesis with TUDCA treatment [115]. However, they did not find any differences in body weight or insulin sensitivity over the three-week duration of the study. Examination of the effects of orally administered TUDCA on insulin sensitivity in obese human subjects revealed a 30% improvement in insulin sensitivity in muscle and liver tissues but no alterations in hepatic TG content were observed [116]. The differences in the mechanism of action between oral treatment and intraperitoneally injected TUDCA may explain some of these contrasting outcomes [115].

 The effectiveness of chemical chaperones such as 4-PBA and TUDCA as a treatment strategy for dyslipidemia, cardiovascular disease, diabetes, and obesity require further study in human subjects. Both 4-PBA and TUDCA have additional functions which may be directly or indirectly alleviating ER stress conditions. Investigation into the discovery of new chemical and biological approaches to enhance ER function and facilitate the trafficking of proteins would be useful for treating ER-stress-related diseases. Furthermore, ways of targeting specific UPR pathways would allow for better specificity in targeting ER stress in various disease states [87]. Currently, small molecules that can target IRE1*α* and alter its endonuclease activity offer hope for further study. These kinase-inhibiting RNase attenuators can also selectively enhance XBP1 mRNA splicing and lead to prevention of apoptotic cell death, while attenuating IRE1*α*-mediated decay of mRNA such as those encoding ER chaperones [117]. The recent finding that unfolded peptides can directly bind to IRE1 and promote its oligomerization and activation suggests that compounds that can target its peptide-binding groove and oligomerization interface may be effective at regulating IRE1 activity [58]. Finally, given the challenges with directly measuring ER stress, assay systems which can assess actual cellular ER stress will prove to be useful [118].

## 7. Conclusions

A growing body of evidence links ER stress and UPR activation to diseases associated with lipid metabolism. The UPR signalling pathways and activation of transcription factors such as XBP1 and ATF6 have novel roles in controlling the transcriptional regulation of lipogenesis. While IRE1*α* itself is protective against ER-stress-induced lipogenesis and hepatic steatosis, its downstream mediator XBP1 promotes transcription of genes involved in fatty acid and cholesterol biosynthesis. Phosphorylation of eIF2*α* downstream of PERK affects the transcriptional activity of C/EBPs, PPAR*γ*, and SREBP-1c thereby leading to lipid accumulation and hepatic steatosis under high-fat-diet conditions. Similar to IRE1*α*, ATF6*α* also protects against ER stress-induced steatosis and lipid droplet formation in mice. Furthermore, nuclear ATF6 attenuates SREBP2-mediated lipogenesis. The exact mechanisms by which ER stress signalling pathways affect lipid homeostasis are incompletely understood. Given the temporal differences in the activation of the three arms of the UPR, a closer examination of each branch of the UPR will allow for a better understanding of how various components of this signalling network impact on lipogenesis and disease progression. Such studies will further enhance our understanding of biological and pharmacological tools needed to effectively treat ER-associated diseases.
